# Depression and anxiety symptoms in internally migrated women and men after the German unification: Baseline results from the German National Cohort Study (NAKO)

**DOI:** 10.1016/j.jmh.2026.100403

**Published:** 2026-02-26

**Authors:** D. Otten, C. Kasinger, L. Kriechel, A.N. Tibubos, K. Berger, G. Schomerus, T. McLaren, M.E. Beutel, S. Speerforck, E. Brähler, NAKO investigators, Heiko Becher, Patricia Bohmann, Hermann Brenner, Stefanie Castell, Daniela Fuhr, Hans J. Grabe, Karin Halina Greiser, Volker Harth, Antje Hebestreit, Jana-Kristin Heise, Stefanie Jaskulski, André Karch, Thomas Keil, Jasmin Kiekert, Lilian Krist, Lena Koch-Gallenkamp, Oliver Kuß, Berit Lange, Michael Leitzmann, Janka Massag, Claudia Meinke-Franze, Rafael Mikolajczyk, Nadia Obi, Annette Peters, Tobias Pischon, Tamara Schikowski, Börge Schmidt, Carsten Oliver Schmidt, Matthias Schulze, Ilais Moreno Velásquez, Kerstin Wirkner

**Affiliations:** 1 Heidelberg Institute of Global Health, Heidelberg University Hospital, Heidelberg, Germany; 2 Department of Epidemiology and Preventive Medicine, University of Regensburg, Regensburg, Germany; 3 Division of Clinical Epidemiology and Aging Research, German Cancer Research Center (DKFZ), Heidelberg, Germany; 4 Helmholtz Centre for Infection Research (HZI), Braunschweig, Germany; 5 Leibniz Institute of Prevention Research and Epidemiology, Department of Prevention and Evaluation, Bremen, Germany; 6 University of Bremen, Health sciences, Bremen; 8 German Center for Neurodegenerative Diseases (DZNE), Greifswald, Germany; 9 Department of Psychiatry and Psychotherapy, University Medicine Greifswald, Germany; 10 German Cancer Research Center (DKFZ) Heidelberg, Division of cancer Epidemiology, Heidelberg, Germany; 11 Institute of Occupational and Maritime Medicine (ZfAM), Universitiy Medical Center Hamburg-Eppendorf, Hamburg, Germany; 12 Leibniz Institute for Prevention Research and Epidemiology – BIPS, Germany; 13 Helmholtz Centre for Infection Research (HZI), Braunschweig, Germany; 14 Institute for Prevention and Cancer Epidemiology, Faculty of Medicine, University of Freiburg, Freiburg, Germany; 15 Institute of Epidemiology and Social Medicine, University of Münster, Münster, Germany; 16 Institute of Social Medicine, Epidemiology and Health Economics, Charité - Universitätsmedizin Berlin, Berlin, Germany; 17 Institute of Clinical Epidemiology and Biometry, University of Würzburg, Würzburg, Germany; 18 State Institute of Health I, Bavarian Health and Food Safety Authority, Erlangen, Germany; 19 Institute for Prevention and Cancer Epidemiology, Faculty of Medicine, University of Freiburg, Freiburg, Germany; 20 Institute of Social Medicine, Epidemiology and Health Economics, Charité - Universitätsmedizin Berlin, Berlin, Germany; 21 German Cancer Research Center, Division of Clinical Epidemiology and Aging Research, Heidelberg; 22 German Diabetes Center (DDZ), Leibniz Center for Diabetes Research, Heinrich Heine University, Düsseldorf, Germany; 23 Helmholtz Centre for Infection Research (HZI), Braunschweig, Germany; 24 Department of Epidemiology and Preventive Medicine, University of Regensburg, Regensburg, Germany; 25 Institute for Medical Epidemiology, Biometrics and Informatics (IMEBI), Interdisciplinary Centre for Health Sciences, Medical Faculty of the Martin Luther University Halle-Wittenberg, Halle (Saale), Germany; 26 Institute for Community Medicine, University Medicine Greifswald, Greifswald, Germany; 27 Institute for Medical Epidemiology, Biometrics and Informatics (IMEBI), Interdisciplinary Centre for Health Sciences, Medical Faculty of the Martin Luther University Halle-Wittenberg, Halle (Saale), Germany; 28 Intsitute of Occupational and Maritime Medicine (ZfAM), Universitiy Medical Center Hamburg-Eppendorf, Hamburg, Germany; 29 Institute of Epidemiology, Helmholtz Zentrum München, German Research Center for Environmental Health (GmbH), Neuherberg, Germany; 30 Chair of Epidemiology, Institute for Medical Information Processing, Biometry and Epidemiology, Medical Faculty, Ludwig-Maximilians-Universität München, Munich, Germany; 31 German Center for Mental Health (DZPG), partner site Munich, Munich, Germany; 32 Max-Delbrück-Center for Molecular Medicine in the Helmholtz Association (MDC), Molecular Epidemiology Research Group, Berlin, Germany; 33 Max-Delbrück-Center for Molecular Medicine in the Helmholtz Association (MDC), Biobank Technology Platform, Berlin, Germany; 34 Charité - Universitätsmedizin Berlin, corporate member of Freie Universität Berlin and Humboldt-Universität zu Berlin, Berlin, Germany; 35 Leibniz Research Institute for Environmental Medicine, Düsseldorf, Germany; 36 Institute for Medical Informatics, Biometry and Epidemiology, University Hospital of Essen, University of Duisburg-Essen, Essen, Germany; 37 Institute for Community Medicine, University Medicine Greifswald, Greifswald, Germany; 38 Department of Molecular Epidemiology, German Institute of Human Nutrition Potsdam-Rehbruecke, Nuthetal, Germany; 39 Institute of nutritional Science, University of Potsdam, Nuthetal, Germany; 40 Max-Delbrück-Center for Molecular Medicine in the Helmholtz Association (MDC), Molecular Epidemiology Research Group, Berlin, Germany; 41 Leipzig Research Centre for Civilization Diseases, University of Leipzig, Leipzig, Germany; a Department of Psychosomatic Medicine and Psychotherapy, University Medical Center of the Johannes Gutenberg University Mainz, Mainz, Germany; b Department of Child and Adolescent Psychiatry, Psychosomatics and Psychotherapy, University Hospital Ulm, Ulm, Germany; c Department of Psychiatry and Psychotherapy, Leipzig University Medical Center, Leipzig, Germany; d Diagnostics in Healthcare and E-Health, University of Trier, Trier, Germany; e Institute of Epidemiology & Social Medicine, University of Muenster, Muenster, Germany; f Department of Medical Psychology and Medical Sociology, University of Leipzig, Leipzig, Germany; g Department of Medical Psychology, University Hospital Jena, Jena, Germany; h Department of Family and Fertility, Federal Institute for Population Research, Wiesbaden, Germany

**Keywords:** Internal migration, Depression and anxiety symptoms, Mental health, German migrants, German unification, NAKO

## Abstract

Background: Internal migration is a special case in Germany, with its history of two formerly divided and re-unified states. In this study, we examined mental health of women and men who migrated internally after the German reunification and compared them with each other and with the non-migrated population in Eastern and Western Germany.

Methods: Baseline data of 161,795 participants (49.9 % women; internal migrants East-West = 7160 [4.4 %] and West-East = 3966 [2.5 %]) from the population-based German National Cohort were used. Internal migration was measured using information on previous (1988) and current residency. To assess mental health (i.e., current depression and anxiety symptoms), the Depression Module of the Patient Health Questionnaire (PHQ-9) and the Generalised Anxiety Disorder Symptoms Scale (GAD-7) were applied. Group differences were assessed using analyses of covariance and Tukey’s Tests. Strengths of effects were tested using omega squared.

Results: Significant differences in levels of current depression and anxiety symptoms between the four groups were found for both women and men, but effect sizes were extremely small (ω² ≈ 0.000–0.002). East-West migrants reported slightly lower current depression symptoms (adj. *M*_women_ = 4.00, adj. *M*_men_ = 3.17) than West-East migrants (adj. *M*_women_ = 4.48, adj. *M*_men_ = 3.69). For East-West migrated men the same was found for anxiety symptoms (adj. M = 2.52 versus adj. M = 2.96). Mental health of internal migrants was not better compared to their non-migrated counterparts.

Conclusion: Subgroup analyses revealed group differences to depend on sex, but show negligible effect sizes, with internal migration only explaining less than 0.2 % of the variance for depression and anxiety symptoms. Internal migration alone is thus no substantial factor in explaining mental health differences. Future studies should use longitudinal data to determine temporal associations between internal migration and mental health.

## Highlights

• Internal German migrants differ from each other regarding sociodemography.
• Differences between internal migrant groups regarding mental health are negligible.
• Substantial heterogeneity within migration groups may dilute average differences.
• Internal migration alone no substantial factor in explaining mental health differences.
• Alignment regarding mental health between East- and West Germany.

## 1 Introduction

### 1.1 Background

Migration has become one of the most pressing issues in society, politics and public health throughout Europe, since approximately 75 million international migrants live in the European Region, amounting to 8.4 % of the total European population (World Health Organization, 2016). This is the highest rate since World War II. Migration processes are accompanied by various challenges, due to changes that take place across different life domains. The adaptation to a new social and cultural environment can be a stimulating experience providing the opportunity for a better life, but it can also be perceived as stressful and challenging. Hence, migration has been portrayed as both a chance and a risk for individuals and societies (Berry et al., 2006).

According to the International Organization for Migration, the term *migrant* is defined as ‘an umbrella term, not defined under international law, reflecting the common lay understanding of a person who moves away from his or her place of usual residence, whether within a country or across an international border, temporarily or permanently, and for a variety of reasons. ”(International Organization for Migration, 2019). Hence, relocation within a country is also a form of migration and referred to as internal migration. Internal migration is, however, a special case of migration (Sieben and Straub, 2018). It cannot be compared directly with migration between countries, since the motives and challenges faced by these migrants differ (e.g., language barriers), especially if migrants had to flee from their countries. However, internal migrants also partly face similar challenges, since they leave their familiar environment behind (Kuo, 2014).

When considering internal migration in recent German history, it is important to note that the country was divided into two separate states in 1949 following the Second World War. In the East, following Soviet occupation, the German Democratic Republic (GDR) was founded, in the West the Federal Republic of Germany (FRG). Nowadays, after the unification in 1990, these represent the Eastern and Western federal states, respectively. The collapse of the GDR and subsequent accession to the FRG brought major economic, cultural, and political changes upon Germany that remained present even 30 years after the unification (Krause, 2019). The 3.8 million people who migrated from East to West Germany between 1991 and 2018 (one fifth to one fourth of the GDR population) (Heller et al., 2020) thus needed to find adaptation mechanisms to function in this new society (Kuo, 2014). This applied to the people migrating in the other direction (a smaller group) to a lesser extent. Therefore, especially migration from East to West Germany may pose a risk for the mental health of these individuals.

Depression and anxiety disorders belong to the most common mental disorders in the Western world (Arias-de la Torre et al., 2021; Simpson et al., 2010). The prevalence of anxiety disorders in populations varies between country and subgroup (Arias-de la Torre et al., 2021; Remes et al., 2016). A systematic review revealed higher prevalence rates of anxiety disorders in women (5.2–8.7 %); young adults (2.5–9.1 %); people with chronic diseases (1.4–70 %); and individuals from Euro/Anglo cultures (3.8–10.4 %) (Remes et al., 2016). The overall prevalence rate of current depression disorder in Europe is 6.4 % (Arias-de la Torre et al., 2021), with the highest rates in Germany and Luxembourg. Based on data of the first 100,000 participants of the German National Cohort Study (NAKO), an overall prevalence rate of 7.8 % for depression (according to the dimensional assessment using the depression module of the Patient Health Questionnaire (PHQ-9); score ≥ 10) (Streit et al., 2023) and 5.2 % for current generalized anxiety symptoms (assessed with the Generalised Anxiety Disorder Symptoms Scale; GAD-7) (Erhardt et al., 2022) was found. Furthermore, in Germany, prevalence of depression and anxiety disorders and symptoms of depression and anxiety are consistently higher in women than in men (Otten et al., 2021).

With regard to mental health of internal German migrants, research results are contradictory. The Saxonian longitudinal study revealed better mental health for migrants moving from East to West Germany compared to non-migrated East Germans (Berth et al., 2014). However, a study using the same data source revealed that this result diminished 30 years after the unification (Kasinger et al., 2021). German representative studies reported more psychological complaints for East to West German migrants (women and men) (Albani et al., 2007) and lower resilience and self-esteem (Beutel et al., 2022) compared to non-migrated East Germans. For internal German migration in the opposite direction (from West to East Germany), a difference was found only for women who reported worse mental health than non-migrated West German women, no difference was found for men (Albani et al., 2007). When focusing on depression results differ. Higher rates of depression were found for internal German migrants in both directions compared to their non-migrated counterparts (Grulke et al., 2004; Otten et al., 2022), whereas another study reported lower levels of depression for West to East German migrants compared to non-migrated West Germans (Albani et al., 2010).

Contrasting results on mental health of internal German migrants from previous studies can be explained by different time points of the studies (i.e., the former Eastern and Western states have aligned over the years),the rather small number of internal German migrants in most studies (especially for West-East German migrants), and combining internal migrants migrating before and after the unification, which is problematic since strong differences exist between the illegal migrations before the unification in 1990 (of which numbers are unknown or vary tremendously) versus the legal migration after the reunification. The large population-based sample of the NAKO study offers the possibility to identify large groups of internal German migrants in both directions who moved after the unification. The aim of the present study was to compare the two groups of internal German migrants with each other and with non-migrated East and West Germans regarding sociodemographic characteristics as well as mental health (i.e., the presence and severity of current depression and anxiety symptoms). This was done for women and men separately.

## 2 Methods

### 2.1 Study design

The NAKO is a population-based cohort study that included 205,415 randomly selected participants aged 20–69 years in 18 regional study centers spread over 13 of the 16 Federal States of Germany (German National Cohort Consortium, 2014). It aims to investigate the causes and risk factors for the development of widespread diseases such as cancer, diabetes, infectious diseases, neurodegenerative/-psychiatric diseases, heart attacks and other major chronic diseases in a long-term cohort design. The present analysis includes data from the baseline survey. Baseline examination included questions on lifestyle, previous illnesses, other health factors, and medication use as well as extraction of biomaterials (e.g., blood sample) and took place between 2014 and 2019. Study participation followed informed consent and was on a voluntary basis. All examinations were performed according to standard operating procedures by certified medical technical assistants. Details of this study can be found elsewhere (German National Cohort Consortium, 2014).

### 2.2 Sample

For this study, we received data of 204,878 participants of the NAKO study (excluding 537 participants [0.3 %] of the total baseline survey). We excluded 16,304 (8.0 %) participants of whom the place of living at time of the baseline study and/or place of living in the year 1988 was unknown. Of the remaining 188,574 participants we excluded 14,893 participants (7.9 %) living abroad in 1988 (international migrants). Further 11,886 persons (6.8 %) with missing values on our outcome variables depression and/or anxiety were excluded. This led to a final sample of *N* = 161,795 participants.

### 2.3 Measures

To assess current depression and anxiety symptoms, the Depression Module of the Patient Health Questionnaire (PHQ-9) (Kocalevent et al., 2013) and the Generalised Anxiety Disorder Symptoms Scale (GAD-7) were applied. Good psychometric properties of the PHQ-9 and GAD-7 scale were found in general German population samples (Hinz et al., 2017; Kliem et al., 2024). Using a Likert scale ranging from 0 = not at all to 3 = nearly every day, participants were asked to indicate how often they were bothered by the respective symptom over the course of the last two weeks. Sum scores ranged from 0 to 27 and from 0 to 21 for depression and anxiety symptoms, respectively. Good internal consistency for both scales was found in our sample (PHQ-9: Cronbach’s α = 0.84, McDonald’s ω = 0.85 and GAD-7: Cronbach’s α =0.85, McDonald’s ω = 0.86).

To assess internal German migration, information on place of residence in 1988 and place of residence at time point of participation in the NAKO study (between 2014 and 2019) were combined. Individuals who lived in the Eastern states in 1988, but were living in the Western states at the time point of their participation in the NAKO study were classified as east-west internal German migrants. Individuals who lived in the Western states in 1988, but were living in the Eastern states at the time point of their participation in the NAKO study were classified as west-east internal German migrants. Persons who lived in East Germany to both time points were labelled as non-migrated East Germans. Persons who lived in West Germany to both time points were labelled as non-migrated West Germans.

Further, age group was categorized in ten-year units based on date of birth and time point of assessment. Partnership was assessed with the question if respondents had a partner (yes/no). Education was based on the International Standard Classification of Education (ISCED-97) levels and further summarized to lower (ISCED-97 level 1/2), intermediate (ISCED-97 level 3/4), and higher (ISCED-97 level 5/6) education, referred to as low, medium, and high education (Dragano et al., 2020). Equivalent income was determined using the monthly net household income and dividing it by the total of equivalence weightings of members of the household according to the OECD-scale (Organisation for Economic Co-operation and Development).

### 2.4 Statistical methods

Descriptive statistics included absolute and relative proportions for categorical data, means, and standard deviations for continuous variables. Additionally, sensitivity analyses were performed.

In order to assess differences between internal German migrants and participants who did not migrate from East to West Germany and vice versa, we performed analyses of covariance (ANCOVA) with PHQ-9 and GAD-7 sum scores as dependent variables and controlling for sociodemographic and economic factors associated with mental health (age, partnership, education, and equivalent income). Since previous studies have shown differences in numbers of women and men migrating from East to West Germany and vice versa, we performed sex stratified analyses. Results of ANCOVA analyses were reported. Tukey’s Tests were performed to correctly interpret significant group differences; estimated group differences and adjusted p-values were reported. Omega squared (ω²) was calculated to test the strength of the ANCOVA effects (Yiğit and Mendes, 2018).

## 3 Results

### 3.1 Participant characteristics

Our sample consisted of *N* = 161,795 participants, including *n* = 106,053 respondents in the non-migrated West German group and *n* = 44,616 respondents in the non-migrated East German group. The group of internal German migrants comprised *n* = 11,126 respondents. Of these, *n* = 3966 migrated from West to East Germany and *n* = 7160 migrated from East to West Germany between 1988 and 2014–2019. The number of women and men was comparable between the non-migrated East-German and West-German group. However, respondents migrating from West to East Germany were predominantly men (58.5 %, *n* = 2321), whereas more women migrated from East to West Germany (54.3 %, *n* = 3887). Although NAKO participants were mostly between 40 and 69 years old by design, East to West German migrants were slightly younger than the respondents in the other groups. In all groups most respondents reported having a partner (80 % or more). The proportion of participants with a high educational level was highest among internal German migrants, especially for respondents migrating from West to East Germany. This group also reported highest equivalent income. Equivalent income was lowest in non-migrated East Germans. Current depression symptoms were reported with a mean value of 3.8 (*SD* = 3.7) and current anxiety symptoms with 3.1 (*SD* = 3.2). Current depression and anxiety symptoms were slightly higher for the internal migration groups compared to their non-migrated counterparts with similar socialization experiences. Further details can be found in Table 1.

**Table 1:** Sociodemographic and current depression and anxiety symptoms of NAKO participants (*N* = 161,795).

	Total (*N* = 161,795)	West-West (*N* = 106,053)	West-East (*N* = 3966)	East-West (*N* = 7160)	East-East (*N* = 44,616)
Sex					
men	81,075 (50.1 %)	53,508 (50.5 %)	2321 (58.5 %)	3273 (45.7 %)	21,973 (49.2 %)
Women	80,720 (49.9 %)	52,545 (49.5 %)	1645 (41.5 %)	3887 (54.3 %)	22,643 (50.8 %)
Age groups					
26–29	3534 (2.2 %)	1852 (1.7 %)	118 (3.0 %)	309 (4.3 %)	1255 (2.8 %)
30–39	18,123 (11.2 %)	10,621 (10.0 %)	450 (11.3 %)	1701 (23.8 %)	5351 (12.0 %)
40–49	45,330 (28.0 %)	28,768 (27.1 %)	1462 (36.9 %)	2663 (37.2 %)	12,437 (29.6 %)
50–59	48,189 (29.8 %)	32,244 (30.4 %)	1191 (30.0 %)	1566 (21.9 %)	13,188 (29.4 %)
60–69	43,144 (26.7 %)	30,006 (28.3 %)	692 (17.4 %)	873 (12.2 %)	11,573 (25.9 %)
70–79	3475 (2.1 %)	2562 (2.4 %	53 (1.3 %)	48 (0.7 %)	812 (1.8 %)
Partner					
No	28,004 (17.3 %)	18,930 (17.9 %)	685 (17.3 %)	1441 (20.2 %)	6948 (15.6 %)
Yes	133,586 (82.7 %)	86,976 (82.1 %)	3274 (82.7 %)	5702 (79.8 %)	37,634 (84.4 %)
Educational level					
Low	2923 (1.9 %)	2563 (2.6 %)	58 (1.6 %)	40 (0.6 %)	262 (0.6 %)
medium	61,771 (41.1 %)	41,667 (42.1 %)	909 (25.1 %)	2339 (35.0 %)	16,856 (40.9 %)
high	85,755 (57.0 %)	54,692 (53.3 %)	2658 (73.3 %)	4311 (64.4 %)	24,094 (58.5 %)
Equivalent income	2383 ± 1482	2534 ± 1582	2648 ± 1721	2398 ± 1357	2007 ± 1130
Current depression symptoms (PHQ-9)	3.84 ± 3.70	3.90 ± 3.74	4.0 ± 3.8	3.78 ± 3.56	3.69 ± 3.60
Current anxiety symptoms (GAD-7)	3.13 ± 3.21	3.17 ± 3.22	3.26 ± 3.29	3.10 ± 3.11	3.04 ± 3.18

*Note.* Descriptive statistics were performed as absolute and relative proportions for categorical data, means, and standard deviations (*M*±*SD*) for continuous variables.

Sociodemographic characteristics stratified for women (*N* = 80,720) and men (*N* = 81,075) are displayed in Supplementary Tables 1 and 2. As mentioned before, West-East German migrants were predominantly men (58.5 %), whereas East-West German migrants were more frequently women (54.3 %). Women who migrated from East to West Germany were slightly younger than non-migrated and West-East German migrated women. Among women, education was highest for both groups of internal German migrants. Among men, level of education was highest in West-East German migrants and lowest in non-migrated East Germans. Women and men who migrated from West to East Germany reported the highest equivalent income. For women, current depression and anxiety symptoms had mean values of 4.3 (*SD* = 3.8) and 3.6 (*SD* = 3.4), respectively. Current depression and anxiety symptoms were highest in women who migrated from West to East Germany compared to all other groups. For men, current depression and anxiety symptoms had mean values of 3.4 (*SD* = 3.5) and 2.7 (*SD* = 3.0), respectively and were highest in the West-East migration group.

### 3.2 Group comparisons between internal German migrants and non-migrated East and West Germans

ANCOVAs revealed significant differences in levels of current depression and anxiety symptoms between the four groups of internal German migrants and non-migrated East and West Germans for women and men, also after including sociodemographic and -economic covariates, but associated effect sizes were negligible [current depression symptoms: *F*_women_(369378) = 11.42, *p* < .001, ω² = 0.0004 and *F*_men_(371433) = 41.99, *p* < .001, ω² = 0.0017; current anxiety symptoms: *F*_women_(369378) = 2.67, *p* = .05, ω² = 0.0001 and *F*_men_(371433) = 37.52, *p* < .001, ω² = 0.0015].

Fig. 1a, Fig. 1b show adjusted mean values of depression- and anxiety symptoms for internally migrated and non-migrated East and West German women. Though ANCOVA tests revealed group differences for women, post hoc comparisons revealed only minimal mean differences. Women who migrated from East to West Germany reported slightly less current depression symptoms compared to non-migrated West German women and women who migrated from West to East Germany. Besides, current depression symptoms were marginally higher among non-migrated West compared to non-migrated East German women. Regarding current anxiety symptoms, no significant differences between the four groups were found, which is consistent with the negligible effect size. Detailed results can be found in Supplementary Tables 3 and 4.

**Fig. 1a:**
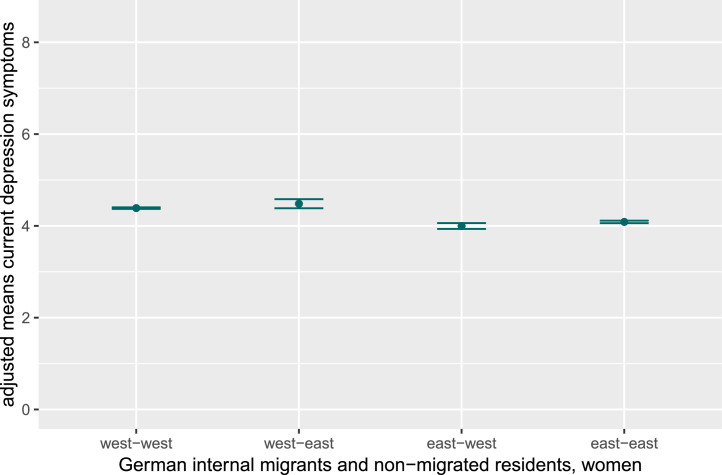
Adjusted mean values for current depression symptoms in women, stratified for internal German migrants and non-migrated East and West Germans.

**Fig. 1b:**
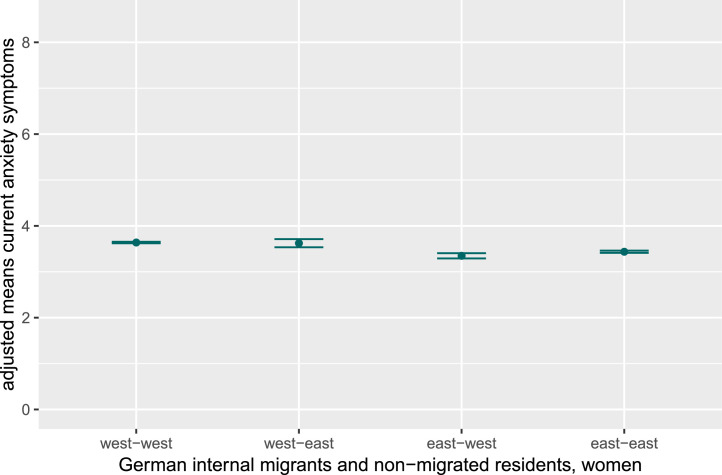
Adjusted mean values for current anxiety symptoms in women, stratified for internal German migrants and non-migrated East and West Germans.

In Fig. 1c, Fig. 1d adjusted mean values of depression- and anxiety symptoms for internally migrated and non-migrated East and West German men are displayed. Also for men the strength of the group differences are marginal. Men who migrated from West to East Germany reported slightly higher values of current depression and anxiety symptoms compared to all other groups. East-west migrated men also reported marginally higher current depression and anxiety symptoms compared to non-migrated East German men. Besides, current depression and anxiety symptoms were marginally higher among non-migrated West compared to non-migrated East German men. Detailed results can be found in Supplementary Tables 5 and 6.

**Fig. 1c:**
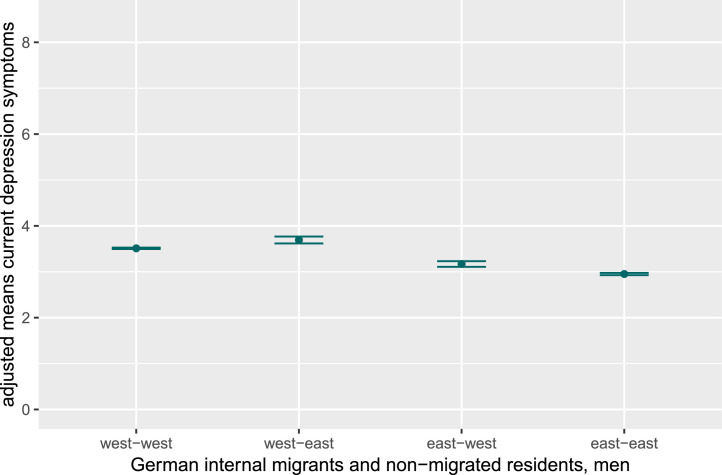
Adjusted mean values for current depression symptoms in men, stratified for internal German migrants and non-migrated East and West Germans.

**Fig. 1d:**
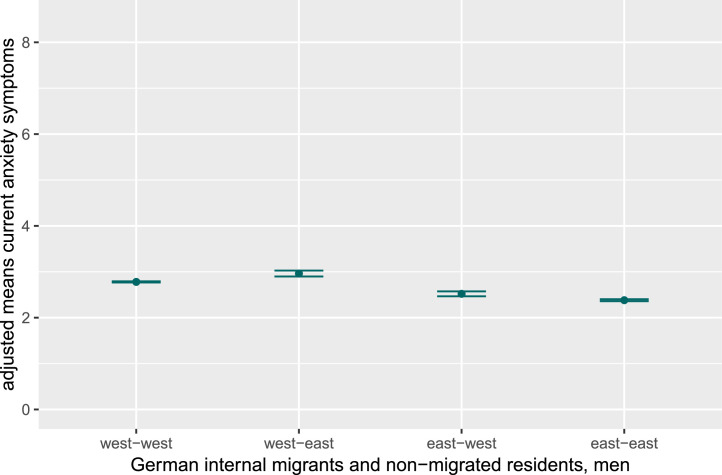
Adjusted mean values for current anxiety symptoms in men, stratified for internal German migrants and non-migrated East and West Germans.

Note: Figures display adjusted mean values and results of post-hoc tests (controlling for age, partnership, education, and equivalent income) for current depression and anxiety symptoms in women and men, stratified for internal German migrants and non-migrated East and West Germans. As effect sizes were small, significant group differences are not indicated in the figures. The y-axis is truncated to highlight data trends, not representing the full range for depression (PHQ-9; range 0–27) and anxiety (GAD-7; range 0–21) symptoms.

## 4 Discussion

### 4.1 Interpretation of the results

The aim of this study was to examine mental health of internal German migrants who moved from East to West Germany or vice versa after the unification of Germany. More specifically, current depression and anxiety symptoms were for women and men separately compared between the two internal German migrant groups and with non-migrated residents in East and West Germany. Although several effects reached statistical significance, all effect sizes were negligible (i.e., ω² ≈ 0.001 for women and ω² ≈ 0.002 for men), indicating that internal migration explained less than respectively 0.1 % and 0.2 % of the variance for current depression and anxiety symptoms in women and men. Internal migration alone is thus no substantial factor in explaining mental health differences. Findings therefore only have limited practical importance.

The small differences between groups can be explained by a high heterogeneity within the groups. Not only had internal migrants different motives for their migration, they also migrated at different time points (early after the unification versus in recent years). Initial mental health benefits due to economic migration level out over time (Kratz, 2020). Furthermore, besides potential benefits, internal migration is also accompanied by stressors. Migrants leave their familiar environment behind (Kuo, 2014) and need to adapt to a new society in which they might experience hostile attitudes and discrimination, which is known to have negative effects on mental health (Pascoe and Smart Richman, 2009). This of course goes for internal migrants in both directions. Overall, the positive and negative aspects of internal migration in Germany might (over time) have approached a near-zero association.

Subgroup level factors influencing mental health partly differ for West to East and East to West internal German migrants. Our results revealed differences in sociodemographic features of both groups. Internal migrants who moved from West to East Germany were mostly men who reported similar mental health (only marginally more current depression and anxiety symptoms) compared to other groups. These men had a high educational level and income. This could point to men who migrated from West to East Germany in order to promote their career by taking over high prestige jobs (Neubecker, 2013) corresponding to a strong socioeconomic position. This is shown to be beneficial for a person’s mental health; it decreases for example the likelihood of persistent or new episodes of depression (Lorant et al., 2003). However, holding leadership positions is also associated with increased stress (Harms et al., 2017) which in turn could decrease one’s mental health. Furthermore, these men migrated presumably in the early years after the unification in a time when East Germany was still adapting towards a changed cultural and political system. During this time, East Germany was an extremely homogeneous society (reinforced by the emigration of specific groups towards the West after the unification) in which countercultures only found little acceptance (Thränhardt, 2008), which made integration difficult and might have had a negative effect on their mental health (Pascoe and Smart Richman, 2009). At the same time the group of West to East migrated men is likely to include men migrating in later years after the unification. Migration from West to East Germany was shown to have increased in recent years, especially by men (Destatis, 2022). This group of men also includes returnees (Jain and Schmithals, 2009) who re-migrated from West to East Germany after having migrated from the GDR to the FRG (migration before the unification) due to family or work reasons (Bundesministerium für Wirtschaft und Energie, 2014). These might be partly retirees whose mental health decreased after transitioning to retirement (Mosconi et al., 2023) and is in line with the general higher age within this group.

In contrast, women made up the majority of the group of East to West migrants. They displayed marginally better mental health (i.e., they reported slightly less current symptoms of depression) than women who migrated the other way around. Women who moved from East to West Germany migrated especially in the first years after the unification and also in the early 2000s. They were younger women (aged 18–30) with a high education level (Martens, 2020; Stawarz et al., 2024). This group most likely migrated for economic reasons, they expected to have a better future in West Germany due to enhanced education and career possibilities, which is often accompanied by better mental health(Barakat and Konstantinidis, 2023). A prior study on internal German migrants also revealed beneficial mental health outcomes for young migrants with high educational attainment (Stawarz et al., 2022). However, for this group of women these advantages might have disappeared over time, since a previous study revealed that increase in women´s well-being after economic migration was not long-lasting (Kratz, 2020).

When comparing internal German migrants with their non-migrated counterparts (e.g., West to East migrated Germans versus non-migrated West Germans), no differences were found for women and a slightly beneficial health, but with negligible effect size for the non-migrated residents was found in men. The healthy migrant hypothesis implying that individuals who are already more resilient and mentally healthier compared to individuals who do not migrate (Chiswick et al., 2008) does thus not seem to apply to the women and men who migrated within Germany, which is in line with a prior study (Holz, 2022). In comparison to prior studies that found differences in mental health between East and West German residents beneficial for East Germans (i.e., less symptoms of depression and anxiety and lower 12-month prevalence of depression (Beutel et al., 2022; Bretschneider et al., 2017; Thom et al., 2017)) the present findings suggest substantially weaker associations in both women and men. This study therefore cannot confirm sex-based patterns as found in other studies (i.e., Otten et al., 2024). Results of this study point to alignment between East and West Germany regarding mental health, which is in line with a study by Entringer et al. revealing no differences between East and West Germany in anxiety and depression symptoms or the number of formal depression diagnoses (Entringer et al., 2024).

### 4.2 Strengths and limitations

Using data from the NAKO study offers the possibility to draw nationwide conclusions on internal German migration, which has been difficult in the past. This large population-based sample moreover allows for subgroup analyses based on region of socialization and current place of living. In this study, we only analyzed internal German migrants who moved after the unification. In previous studies, internal German migrants relocating before and after the unification were often mixed. It is, however, important to distinguish these groups, since the migration before unification was mostly forced migration and thus not comparable to migration after the unification in which persons migrated by choice.

Several limitations have to be mentioned. First, although we know in this study that internal migration took place after the unification, the exact time point of migration was unknown. Therefore, we could not separate respondents who migrated a long time ago and already lived in East or West Germany for a longer time period from the ones who migrated more recently and we do not know whether respondents migrated more frequently between East and West Germany. Furthermore, information on reason for migration, that might differ substantially regarding time point of migration and between women and men, was not available in this study. This might be important for the current mental health state. Negative consequences of migration might be replaced with positive experiences when the migration lies further in the past. Second, a potential selection effect of migration could be present. Opposite to the healthy migrant hypothesis, it is also possible that people with mental ill-health might be more likely to move to leave detrimental surroundings and herewith to improve their health. However, as cross-sectional data was used, mental health before migration could not be examined and no temporal and causal pathways could be tested. Third, the fact that not all of Germany’s 16 Federal states were represented in the sample is another shortcoming for the generalizability of the results. Future studies should include the exact time point of internal migration as well as its main migration motive. Herewith different time periods in which migration took place and its associations with mental health can be studied.

### 4.3 Conclusion

Though this study finds significant differences in levels of current depression and anxiety symptoms between groups of internal German migrants and non-migrated East and West Germans in both women and men, effects sizes were extremely small with internal migration only explaining 0.1–0.2 % of the variance for depression and anxiety symptoms. Internal migration alone is thus no substantial factor in explaining mental health differences. This is possibly due to a larger within-group variation than between-group variation. For both women and men, the groups of internal migrants most likely exist of highly distinctive subgroups including individuals who migrated at different time points (in the first years after the unification versus in recent years) and with different motives. Advantages and disadvantages of internal migration might (over time) lead to a near-zero net association. Our results further point to alignment regarding mental health between East- and West Germany. These results enrich the discussion of the contradictory findings on internal German migration and its mental health consequences. Future studies should apply longitudinal data to assess time point of migration, temporal changes and focus on regional macro characteristics that might impact residents’ mental health.

## Ethics approval and consent to participate

The *German National Cohort/ Nationale Gesundheitsstudie* is conducted with the approval of the respective ethics committees of the study centres and is in accordance with national law and with the Declaration of Helsinki of 1975 (in the current, revised version).

## Consent for data utilisation

Written informed consent was obtained from all participants.

## Availability of data

Access to and use of *NAKO* data and biosamples can be obtained via an electronic application portal (https://transfer.nako.de).

## Funding

This research was spported by the German Federal Ministry of Education and Research (grant number 01UJ1911AY). This funding source had no other role other than financial support. This study was conducted using data from the *German National* Cohort (Nationale Gesundheitsstudie; www.nako.de). The *German National Cohort* is funded by the *Federal Ministry of Education and Research* (project funding reference numbers: 01ER1301A/B/C, 01ER1511D, 01ER1801A/B/C/D and 01ER2301A/B/C), federal states of Germany and the *Helmholtz Association*, the participating universities and the institutes of the *Leibniz Association*.

## CRediT authorship contribution statement

**D. Otten:** Conceptualization, Methodology, Software, Validation, Formal analysis, Investigation, Data curation, Writing – original draft, Visualization, Project administration. **C. Kasinger:** Conceptualization, Methodology, Software, Validation, Writing – original draft. **L. Kriechel:** Methodology, Validation, Writing – original draft. **A.N. Tibubos:** Conceptualization, Writing – original draft, Funding acquisition. **K. Berger:** Conceptualization, Writing – review & editing. **G. Schomerus:** Resources, Writing – review & editing. **T. McLaren:** Validation, Writing – original draft. **M.E. Beutel:** Conceptualization, Resources, Writing – review & editing, Supervision, Project administration, Funding acquisition. **S. Speerforck:** Conceptualization, Writing – review & editing, Supervision. **E. Brähler:** Conceptualization, Resources, Writing – review & editing, Supervision, Funding acquisition.

## Declaration of competing interest

The authors declare the following financial interests/personal relationships which may be considered as potential competing interests:

HJG has received travel grants and speakers´ honoraria from Neuraxpharm, Servier, Indorsia and Janssen Cilag. The other authors declare that they have no competing interests.

## Appendix Supplementary materials

Image, application 1
