# Supplementary material for: Depression and anxiety symptoms in internally migrated women and men after the German unification: Baseline results from the German National Cohort Study (NAKO)
Source: J Migr Health. 2026 Feb 26;13:100403. doi: 10.1016/j.jmh.2026.100403 (PMC13010428; doi:10.1016/j.jmh.2026.100403)
Supplement: Supplementary file 1 [file mmc1.docx]

**Supplementary Table 1.** *Sociodemographic and current depression and anxiety symptoms of women (N = 80,720) stratified for internal German migrants and non-migrated respondents.*

|  | Total  (*N* = 80,720) | West-West (*N* = 52,545) | West-East (*N* = 1,645) | East-West (*N* = 3,887) | East-East (*N* = 22,643) |  |
| --- | --- | --- | --- | --- | --- | --- |
| Age groups |  |  |  |  |  |  |
| 20-29 | 1,836 (2.3%) | 969 (1.8%) | 54 (3.3%) | 168 (4.3%) | 645 (2.8%) |  |
| 30-39 | 9,120 (11.3%) | 5,293 (10.1%) | 237 (14.4%) | 929 (23.9%) | 2,661(11.8%) |  |
| 40-49 | 22,591 (28.0%) | 14,144 (26.9%) | 611 (37.1%) | 1,482 (38.1%) | 6,354 (28.1%) |  |
| 50-59 | 24,332 (30.1%) | 16,263 (31.0%) | 476 (28.9%) | 822 (21.1%) | 6,771 (29.9%) |  |
| 60-69 | 21,252 (26.3%) | 14,678 (27.9%) | 249 (15.1%) | 467(12.0%) | 5,858 (25.9%) |  |
| 70-79 | 1,589 (2.0%) | 1,198 (2.3%) | 18 (1.1%) | 19 (0.5%) | 354 (1.1%) |  |
| Partner |  |  |  |  |  |  |
| no | 16,552 (20.5%) | 11,378 (21.7%) | 356 (21.7%) | 826 (21.3%) | 3,992 (17.6%) |  |
| yes | 64,063 (79.5%) | 41,096 (78.3%) | 1,287 (78.3%) | 3,052 (78.7%) | 18,628 (82.4%) |  |
| Educational level |  |  |  |  |  |  |
| low | 1,912 (2.5%) | 1,708 (3.5%) | 30 (2.0%) | 25 (0.7%) | 149 (0.7%) |  |
| medium | 34,819 (46.4%) | 24,802 (50.6%) | 462 (30.6%) | 1,295 (35.7%) | 8,260 (39.6%) |  |
| high | 38,256 (51.0%) | 22,498 (45.9%) | 1,016 (67.4%) | 2,303 (63.6%) | 12,439 (59.7%) |  |
| Equivalent income | 2,242±1,322 | 2,373±1,412 | 2,450±1,528 | 2,319±1,325 | 1,925±1,009 |  |
| Current depression symptoms (PHQ-9) | 4.3±3.82 | 4.35±3.85 | 4.43±3.84 | 4.14±3.69 | 4.22±3.76 |  |
| Current anxiety symptoms (GAD-7) | 3.58±3.38 | 3.59±3.38 | 3.61±3.37 | 3.46±3.27 | 3.57±3.39 |  |

*Note.* Descriptive statistics were performed as absolute and relative proportions for categorical data, means, and standard deviations (*M*±*SD*) for continuous variables.

**Supplementary Table 2.** *Sociodemographic and current depression and anxiety symptoms of men (N = 81,075) stratified for internal German migrants and non-migrated respondents.*

|  | Total  (*N* = 81,075) | West-West (*N* = 53,508) | West-East (*N* = 2,321) | East-West (*N* = 3,273) | East-East (*N* = 21,973) |  |
| --- | --- | --- | --- | --- | --- | --- |
| Age groups |  |  |  |  |  |  |
| 20-29 | 1,698 (2.1%) | 883 (1.7%) | 64 (2.8%) | 141 (4.3%) | 610 (2.8%) |  |
| 30-39 | 9,003 (11.1%) | 5,328 (10.0%) | 213 (9.2%) | 772 (23.6%) | 2,690 (12.2%) |  |
| 40-49 | 22,739 (28.0%) | 14,624 (27.3%) | 851 (36.7%) | 1,181 (36.1%) | 6,083 (27.7%) |  |
| 50-59 | 23,857 (29.4%) | 15,981 (29.9%) | 715 (30.8%) | 744 (22.7%) | 6,417 (29.2%) |  |
| 60-69 | 21,892 (27.0%) | 15,328 (28.6%) | 443 (19.1%) | 406 (12.4%) | 5,715 (26.0%) |  |
| 70-79 | 1,886 (2.3%) | 1,364 (2.5%) | 35 (1.5%) | 29 (0.9%) | 458 (2.1%) |  |
| Partner |  |  |  |  |  |  |
| no | 11,452 (14.1%) | 7,552 (14.1%) | 329 (14.2%) | 615 (18,8%) | 2,956 (13.5%) |  |
| yes | 69,523 (85.9%) | 45,880 (85.9%) | 1,987 (85.8%) | 2,650 (81.2%) | 19,006 (86.5%) |  |
| Educational level |  |  |  |  |  |  |
| low | 1,011 (1,3%) | 855 (1.7%) | 28 (1.3%) | 15 (0.5%) | 113 (0.6%) |  |
| medium | 26,952 (35.7%) | 16,865 (33.8%) | 447 (21.1%) | 1,044 (34.0%) | 8,596 (42.2%) |  |
| high | 47,499 (62.9%) | 32,194 (64.5%) | 1,642 (77.6%) | 2,008 (65.5%) | 11,655 (57.2%) |  |
| Equivalent income | 2,519±1611 | 2,687±1,715 | 2,703±1,830 | 2,490±1,388 | 2,093±1,236 |  |
| Current depression symptoms (PHQ-9) | 3.39±3.51 | 3.47±3.58 | 3.7±3.73 | 3.35±3.35 | 3.16±3.33 |  |
| Current anxiety symptoms (GAD-7) | 2.69±2.96 | 2.76±3.00 | 3.02±3.21 | 2.67±2.85 | 2.5±2.84 |  |

*Note.* Descriptive statistics were performed as absolute and relative proportions for categorical data, means, and standard deviations (*M*±*SD*) for continuous variables.

**Supplementary Table 3.** *Adjusted mean values for current depression and anxiety symptoms for internal German migrants and non-migrated East- and West Germans after controlling for sociodemographic and -economic factors, stratified for women.*

|  | Adj. mean | 95% CI  lower bound | 95% CI  upper bound |
| --- | --- | --- | --- |
| ***Current depression symptoms*** |  |  |  |
| West Germans | 4.39 | 4.35 | 4.42 |
| West-East internal migrants | 4.48 | 4.29 | 4.68 |
| East-West internal migrants | 4.00 | 3.87 | 4.12 |
| East Germans | 4.09 | 4.03 | 4.14 |
| ***Current anxiety symptoms*** |  |  |  |
| West Germans | 3.64 | 3.61 | 3.67 |
| West-East internal migrants | 3.62 | 3.45 | 3.80 |
| East-West internal migrants | 3.35 | 3.24 | 3.46 |
| East Germans | 3.44 | 3.39 | 3.48 |

*Note.* Adjusted mean values with corresponding confidence interval are reported. Current depression symptoms range from 0 to 27, current anxiety symptoms range from 0 to 21. All ω² values indicate negligible effect sizes.

**Supplementary Table 4.** *Differences in means for current depression and anxiety symptoms for internal German migrants and non-migrated East- and West Germans after controlling for sociodemographic and -economic features, stratified for women.*

|  | Mean group difference | 95% CI  lower bound | 95% CI  upper bound | Adjusted  *p*-value |
| --- | --- | --- | --- | --- |
| ***Depression*** |  |  |  |  |
| West-West versus West-East | 0.10 | -0.15 | 0.36 | *p* = 0.733 |
| West-West versus East-West | 0.25 | 0.08 | 0.41 | *p* = 0.001 |
| West-West versus East-East | 0.15 | 0.07 | 0.23 | *p* < 0.001 |
| West-East versus East-West | 0.35 | 0.05 | 0.65 | *p* = 0.015 |
| West-East versus East-East | 0.25 | -0.01 | 0.51 | *p* = 0.066 |
| East-East versus East-West | 0.10 | -0.08 | 0.27 | *p* =0.485 |
| ***Anxiety*** |  |  |  |  |
| West-West versus West-East | 0.04 | -0.19 | 0.27 | *p* = 0.979 |
| West-West versus East-West | 0.14 | -0.01 | 0.29 | *p* = 0.073 |
| West-West versus East-East | 0.05 | -0.03 | 0.12 | *p* = 0.339 |
| East-West versus West-East | 0.18 | -0.09 | 0.45 | *p* = 0.325 |
| East-East versus West-East | 0.08 | -0.15 | 0.32 | *p* = 0.802 |
| East-East versus East-West | 0.10 | -0.06 | 0.25 | *p* = 0.406 |

*Note.* Differences in mean values between internal German migrants and non-migrated East and West Germans with corresponding confidence interval are reported. All ω² values indicate negligible effect sizes.

**Supplementary Table 5.** *Adjusted mean values for current depression and anxiety symptoms for internal German migrants and non-migrated East- and West Germans after controlling for sociodemographic- and economic factors, stratified for men.*

|  | Adj. mean | 95% CI  lower bound | 95% CI  upper bound |
| --- | --- | --- | --- |
| ***Current depression symptoms*** |  |  |  |
| West Germans | 3.51 | 3.48 | 3.54 |
| West-East internal migrants | 3.69 | 3.55 | 3.84 |
| East-West internal migrants | 3.17 | 3.05 | 3.29 |
| East Germans | 2.95 | 2.90 | 3.00 |
| ***Current anxiety symptoms*** |  |  |  |
| West Germans | 2.78 | 2.75 | 2.80 |
| West-East internal migrants | 2.96 | 2.83 | 3.09 |
| East-West internal migrants | 2.52 | 2.41 | 2.62 |
| East Germans | 2.38 | 2.34 | 2.42 |

*Note.* Adjusted mean values with corresponding confidence interval are reported. Current depression symptoms range from 0 to 27, current anxiety symptoms range from 0 to 21. All ω² values indicate negligible effect sizes.

**Supplementary Table 6.** *Differences in means for current depression and anxiety symptoms for internal German migrants and non-migrated East- and West Germans after controlling for sociodemographic and -economic features, stratified for men.*

|  | Mean group difference | 95% CI  lower bound | 95% CI  upper bound | Adjusted  *p*-value |
| --- | --- | --- | --- | --- |
| ***Depression*** |  |  |  |  |
| West-East versus West-West | 0.20 | 0.01 | 0.40 | *p* = 0.039 |
| West-West versus East-West | 0.09 | -0.08 | 0.25 | *p* = 0.528 |
| West-West versus East-East | 0.30 | 0.23 | 0.37 | *p* < 0.001 |
| West-East versus East-West | 0.29 | 0.04 | 0.53 | *p* = 0.015 |
| West-East versus East-East | 0.50 | 0.30 | 0.70 | *p* < 0.001 |
| East-West versus East-East | 0.21 | 0.04 | 0.38 | *p* = 0.007 |
| ***Anxiety*** |  |  |  |  |
| West-East versus West-West | 0.23 | 0.06 | 0.39 | *p* = 0.003 |
| West-West versus East-West | 0.06 | -0.08 | 0.20 | *p* = 0.713 |
| West-West versus East-East | 0.23 | 0.17 | 0.30 | *p* < 0.001 |
| West-East versus East-West | 0.29 | 0.07 | 0.50 | *p* = 0.003 |
| West-East versus East-East | 0.46 | 0.30 | 0.63 | *p* < 0.001 |
| East-West versus East-East | 0.18 | 0.03 | 0.32 | *p* = 0.010 |

*Note.* Differences in mean values between internal German migrants and non-migrated East and West Germans with corresponding confidence interval are reported. All ω² values indicate negligible effect sizes.
